# Evolution and Cellular Function of Monothiol Glutaredoxins: Involvement in Iron-Sulphur Cluster Assembly

**DOI:** 10.1002/cfg.406

**Published:** 2004-06

**Authors:** Felipe Vilella, Rui Alves, María Teresa Rodríguez-Manzaneque, Gemma Bellí, Swarna Swaminathan, Per Sunnerhagen, Enrique Herrero

**Affiliations:** 1 Departament de Ciències Mèdiques Bàsiques Facultat de Medicina Universitat de Lleida Rovira Roure 44 Lleida 25198 Spain; 2 Department of Cell and Molecular Biology Göteborg University PO Box 462 Göteborg S-40530 Sweden

## Abstract

A number of bacterial species, mostly proteobacteria, possess monothiol glutaredoxins homologous to the *Saccharomyces cerevisiae* mitochondrial protein Grx5, which is involved in iron–sulphur cluster synthesis. Phylogenetic profiling is used to predict that bacterial monothiol glutaredoxins also participate in the iron–sulphur cluster (ISC) assembly machinery, because their phylogenetic profiles are similar to the profiles of the bacterial homologues of yeast ISC proteins. High evolutionary co-occurrence is observed between the Grx5 homologues and the homologues of the Yah1 ferredoxin, the scaffold proteins Isa1 and Isa2, the frataxin protein Yfh1 and the Nfu1 protein. This suggests that a specific functional interaction exists between these ISC machinery proteins. Physical interaction analyses using low-definition protein docking predict the formation of strong and specific complexes between Grx5 and several components of the yeast ISC machinery. Two-hybrid analysis has confirmed the *in vivo* interaction between Grx5 and Isa1. Sequence comparison techniques and cladistics indicate that the other two monothiol glutaredoxins of *S. cerevisiae*, Grx3 and Grx4, have evolved from the fusion of a thioredoxin gene with a monothiol glutaredoxin gene early in the eukaryotic lineage, leading to differential functional specialization. While bacteria do not contain these chimaeric glutaredoxins, in many eukaryotic species Grx5 and Grx3/4-type monothiol glutaredoxins coexist in the cell.


**Comparative and Functional Genomics**
*Comp Funct Genom* 2004; **5**: 328–341.
Published online in Wiley InterScience (www.interscience.wiley.com). **DOI:** 10.1002/cfg.406

Research Paper

### Evolution and cellular function of monothiol glutaredoxins: involvement in iron–sulphur cluster assembly

Felipe Vilella[1], Rui Alves[1], María Teresa Rodríguez-Manzaneque[1], Gemma Bellí[1], Swarna Swaminathan[2], Per Sunnerhagen[2] and Enrique Herrero[1]*
[1]*Departament de Ciències Mèdiques Bàsiques, Facultat de Medicina, Universitat de Lleida, Rovira Roure 44, 25198-Lleida, Spain*
[2]*Department of Cell and Molecular Biology, Göteborg University, PO Box 462, S-40530 Göteborg, Sweden*

**Correspondence to:*
*Enrique Herrero, Dept. de Ciencies Mediques Basiques, Facultat de Medicina, Universitat de Lleida, Rovira Roure 44, 25198- Lleida, Spain.*
*E-mail: enric.herrero@cmb.udl.es*

### Abstract

**A number of bacterial species, mostly proteobacteria, possess monothiol glutaredoxins homologous to the *Saccharomyces cerevisiae* mitochondrial protein Grx5, which is involved in iron–sulphur cluster synthesis. Phylogenetic profiling is used to predict that bacterial monothiol glutaredoxins also participate in the iron–sulphur cluster (ISC) assembly machinery, because their phylogenetic profiles are similar to the profiles of the bacterial homologues of yeast ISC proteins. High evolutionary co-occurrence is observed between the Grx5 homologues and the homologues of the Yah1 ferredoxin, the scaffold proteins Isa1 and Isa2, the frataxin protein Yfh1 and the Nfu1 protein. This suggests that a specific functional interaction exists between these ISC machinery proteins. Physical interaction analyses using low-definition protein docking predict the formation of strong and specific complexes between Grx5 and several components of the yeast ISC machinery. Two-hybrid analysis has confirmed the *in vivo* interaction between Grx5 and Isa1. Sequence comparison techniques and cladistics indicate that the other two monothiol glutaredoxins of *S. cerevisiae*, Grx3 and Grx4, have evolved from the fusion of a thioredoxin gene with a monothiol glutaredoxin gene early in the eukaryotic lineage, leading to differential functional specialization. While bacteria do not contain these chimaeric glutaredoxins, in many eukaryotic species Grx5 and Grx3/4-type monothiol glutaredoxins coexist in the cell.**
**Copyright © 2004 John Wiley & Sons, Ltd.**

Received: 11 November 2003
Accepted: 2 March 2004

**Keywords:** **glutaredoxin; mitochondria; oxidative stress; phylogenetic profile; protein docking; thioredoxin**

### Introduction

Glutaredoxins are thiol oxidoreductases that regulate the redox status of protein sulphydryl groups using glutathione as hydrogen donor (Holmgren, 1989). They differ from thioredoxins in that the latter use NADH as hydrogen donor via thioredoxin reductase. Dithiol glutaredoxins are small proteins, with a conserved two-cysteine active site, that catalyse the reduction of disulphide bonds in a two-step reaction involving an intermediate mixed disulphide between the target protein and the glutaredoxin molecule (Bushweller *et al*., 1992; Holmgren and Aslund, 1995). *Saccharomyces cerevisiae* cells contain two cytoplasmic dithiol glutaredoxins, Grx1 and Grx2, which are involved in the defence against oxidative stress (Luikenhuis *et al*., 1997). They are not required for normal cell growth, except in the absence of the two cytoplasmic thioredoxins, Trx1 and Trx2 (Draculic *et al*., 2000). More recently, a family of three monothiol glutaredoxins (Grx3, Grx4 and Grx5) has been described in *S. cerevisiae* (Rodríguez-Manzaneque *et al*., 1999). They have an active site with a single

Copyright © 2004 John Wiley & Sons, Ltd.

cysteine (Cys–Gly–Phe–Ser) that is conserved in homologous proteins identified in many organisms that range from bacteria to humans (Bellí *et al.*, 2002). These monothiol glutaredoxins could participate in the deglutathionylation of mixed disulphides formed between sulphydryl groups in the target protein and the cysteinyl residue of glutathione, using the single cysteine residue at the active site (Rahlfs *et al.*, 2001; Herrero and Ros, 2002).

Of the three monothiol glutaredoxins in yeast, only Grx5 has been assigned a function in the cell. It participates in the formation of iron–sulphur (Fe–S) clusters in the mitochondria (Rodríguez-Manzaneque *et al.*, 2002), together with the matrix mitochondrial proteins Nfs1 (cysteine desulphurase), Isu1/Isu2, Isa1/Isa2, Ssq1 and its co-chaperone Jac1, Yah1 (ferredoxin), Arh1 (ferredoxin reductase), Nfu1 and Yfh1 (reviewed in Lill and Kispal, 2000; Mühlenhoff and Lill, 2000; see also Mühlenhoff *et al.*, 2002). The ABC transporter Atm1 (localized at the mitochondrial inner membrane) and the intermembrane space protein Erv1 participate in the export of Fe–S clusters for extramitochondrial proteins. All the above proteins constitute the so-called ISC (iron–sulphur cluster assembly) machinery.

Phylogenetic profiling, i.e. the pattern of co-occurrence of genes across genomes, is a tool that allows predictions to be made about the functional relationship of the respective protein products (Gaasterland and Ragan 1998; Pellegrini *et al.*, 1999). Groups of genes that are involved in the same or related functions, or are part of the same cellular structure, tend to co-evolve and to have homologues in the same subset of organisms. Other types of genomic context information can also be used for computational prediction of protein function, such as the fusion of genes into a single open reading frame, the conservation of gene order, the co-occurrence of genes in potential bacterial operons, or the co-occurrence of protein products in the same eukaryotic cell compartment (Marcotte *et al.*, 1999, 2000; Huynen *et al.*, 2000). The evolutionary co-occurrence of genes can help to discriminate between subsets of protein products sharing a general function, and to establish different subprocesses in which members of these subsets would display stronger functional relationships and/or even establish physical interactions (Huynen *et al.*, 2000; Ettema *et al.*, 2001). Phylogenetic profiling (in parallel with biochemical approaches; Chen *et al.*, 2000; Mühlenhoff *et al.*, 2002) has been used to determine that the yeast frataxin protein Yfh1 has a direct role in Fe–S cluster assembly (Huynen *et al.*, 2001), because prokaryotic orthologues of Yfh1 have a similar profile to orthologues of Jac1, Isa1/2 and Yah1.

In this work we determine the phylogenetic profile of Grx5 and its prokaryotic homologues, and compare it with the profiles of other proteins participating in Fe–S cluster synthesis, in order to establish the subset of proteins that display a more similar profile to Grx5. We also make a computational analysis of potential physical interactions between Grx5 and other ISC proteins, which is paralleled by studies of *in vivo* interactions. These data are analysed to specify the functional role of Grx5 in Fe–S cluster biosynthesis. Additionally, we present evidence for the fusion of a thioredoxin gene with a monothiol glutaredoxin gene early in the eukaryotic lineage, leading to differential functional specialization between Grx5 and Grx3/Grx4.

### Materials and methods

For homology comparisons of the individual proteins shown in Figure 3, PSI-BLAST (version 2.2.4) searches (Altschul *et al.*, 1997) were done (three iterations, $E = 0.001$), using the National Center of Biotechnology Information (NCBI) GenBank database (Benson *et al.*, 2001; August 2002 version). Positive sequences were inspected manually for the presence of residues that are essential for the biological activity of the proteins, in cases where these were known. Sequences lacking these residues were considered non-homologous.

Multiple sequence alignments were carried out with the ClustalW programme (Thompson *et al.*, 1994). Internal gaps were not eliminated, and the Blosum80 matrix option was used for alignment. ClustalW alignments were bootstrapped 1000 times and, using maximum parsimony, phylogenetic trees were calculated for the bootstrapped sets. Majority consensus trees were built from these sets and are presented in Figures 1C and 2. The treatment of ClustalW alignments to obtain the consensus trees was done using the PHYLIP suite of programmes (Felsenstein, 1993).

Prediction for protein mitochondrial location was done using the Mitoprot programme (Claros and

Copyright © 2004 John Wiley & Sons, Ltd. 

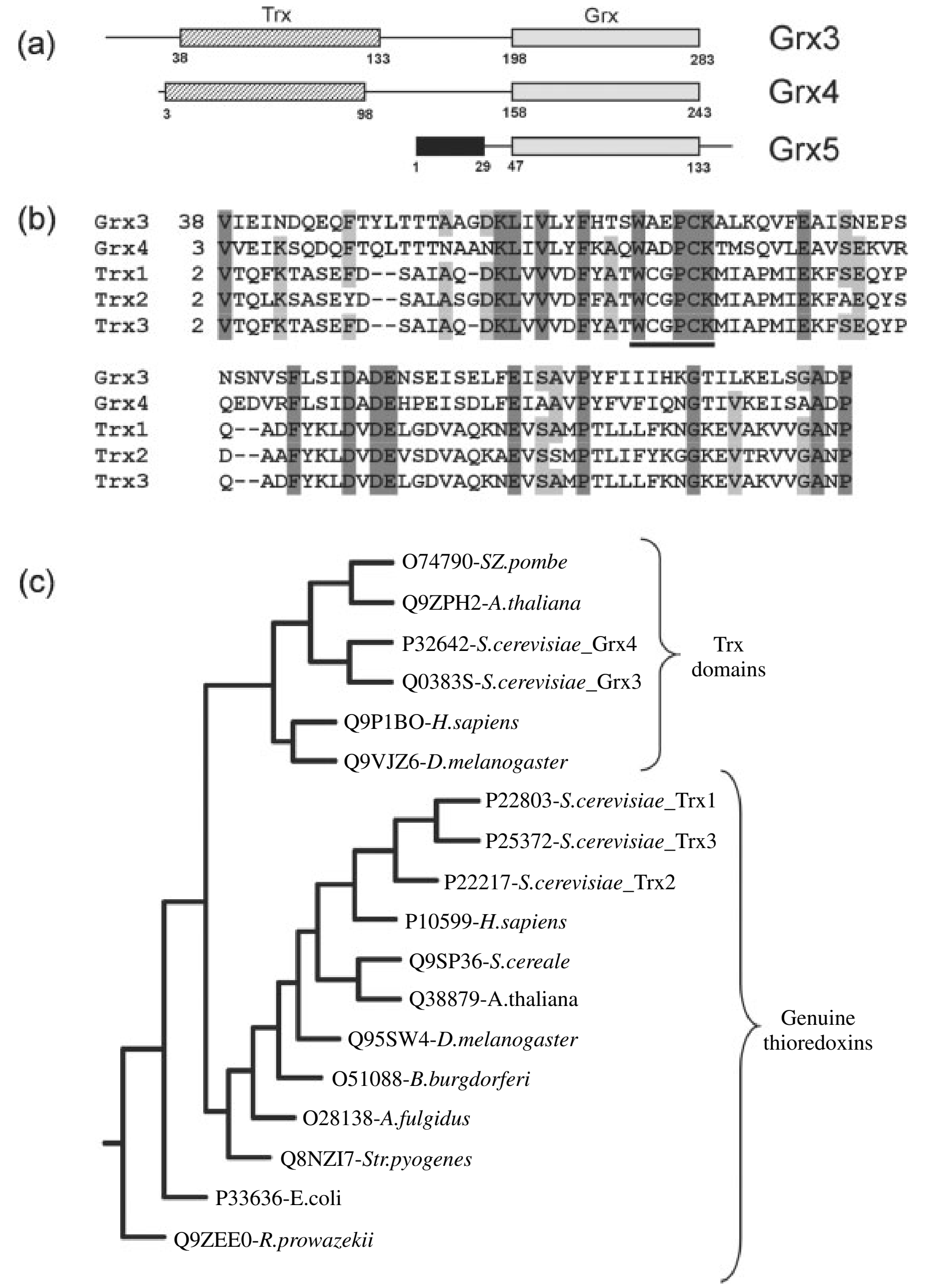

**Figure 1.** Structural comparison between the three monothiol glutaredoxins of *S. cerevisiae* (Grx3, Grx4 and Grx5). (A) Localization of the thioredoxin-like (Trx) and monothiol glutaredoxin (Grx) domains. The mitochondrial targeting sequence of Grx5 is indicated as a black box. Numbers show the positions of the amino acids that limit the respective domains. (B) ClustalW alignments of the Trx domains of Grx3 and Grx4 and the three genuine thioredoxins of *S. cerevisiae* (Trx1, Trx2 and Trx3). Non-homologous flanking regions are not shown. Conserved residues are shaded. The horizontal line indicates the position of the thioredoxin active site. (C) Phylogenetic tree resulting from maximum parsimony analysis of bootstrapped ClustalW alignments of a number of Trx domains of monothiol glutaredoxins and of genuine thioredoxins from the indicated organisms. SwissProt entries of the respective proteins are shown. Species names: *Schizosaccharomyces pombe, Arabidopsis thaliana, Saccharomyces cerevisiae, Homo sapiens, Drosophila melanogaster, Secale cereale, Borrelia burgdorferi, Archaeoglobus fulgidus, Streptococcus pyogenes, Escherichia coli, Rickettsia prowazekii*

Copyright © 2004 John Wiley & Sons, Ltd. 

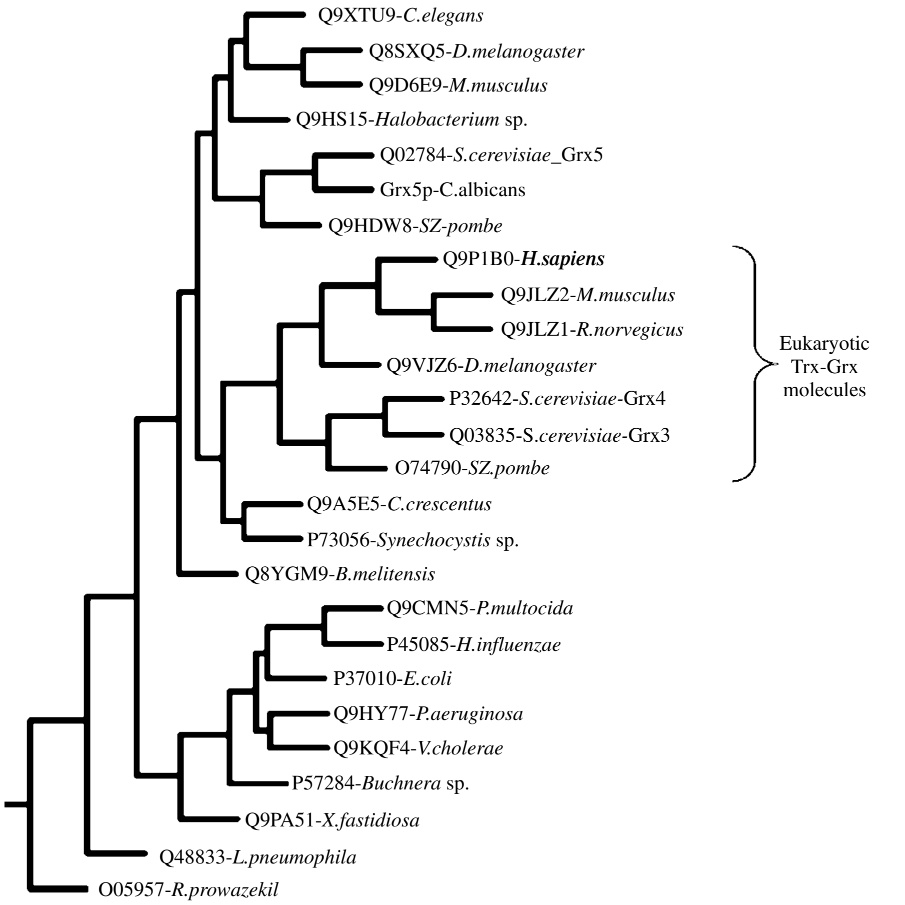

**Figure 2.** Phylogenetic tree of *S. cerevisiae* Grx5 glutaredoxin and a number of homologues from prokaryotic and eukaryotic organisms, obtained using maximum parsimony analysis of bootstrapped ClustalW alignments of the indicated proteins (SwissProt entries are shown, except for the *C. albicans* Grx5 homologue). Names in bold type correspond to predicted mitochondrial proteins. Alignments were done with the Grx glutaredoxin domain without adjacent sequences. Molecules with the Trx–Grx structure are within the bracket. The other proteins in the tree lack the Trx domain. Species names: *Caenorhabditis elegans, Drosophila melanogaster, Mus musculus, Halobacterium* sp., *Saccharomyces cerevisiae, Candida albicans, Schizosaccharomyces pombe, Homo sapiens, Rattus norvegicus, Caulobacter crescentus, Synechocystis* sp., *Brucella melitensis, Pasteurella multocida, Haemophilus influenzae, Escherichia coli, Pseudomonas aeruginosa, Vibrio cholerae, Buchnera* sp., *Xylella fastidiosa, Legionella pneumophila, Rickettsia prowazekii*

Vincens, 1996). Only proteins with scores higher than 0.8 were considered positive.

Whole-proteome phylogenetic profiling was done using proteomes from the Institute for Chemical Research of Kyoto University (KEEG) and GenBank databases. The proteome of all organisms described in the KEGG database (version 23.0) was completed with information from the GenBank database (August 2002 version). Homology searches for each of the *S. cerevisiae* proteins in each of the other proteomes was done running version 2.2.4 of PSI-BLAST locally, with parameters $E = 0.001$ and three iterations. Taking as reference the vector for the Grx5 protein (or its *Buchnera* homologue), we calculated the index of co-occurrence for all proteins as given by:

$$\mathrm{CI} = \Sigma\delta_{\mathrm{ij-iGrx5}}/\text{total number of organisms}$$

where $\delta_{\mathrm{ij-Grx5}}$ is the Kronecker delta function, taken to be 1 if Grx5 and protein j both have (or do not have) homologues in the proteome of organism i, and 0 otherwise.

Models for the three-dimensional structure of the proteins of interest have been obtained using 3D-JIGSAW (Bates *et al.*, 2001). The docking

Copyright © 2004 John Wiley & Sons, Ltd. 

experiments were then performed using GRAMM (Vakser and Jiang, 2001).

Two-hybrid analyses were carried out as described in Rodríguez-Navarro *et al.* (2002), using a pGBT9 derivative that expressed the Gal4 (DNA binding domain)–Grx5 fusion and a number of pACT2 derivatives that expressed the Gal4 (activation domain) fused to individual ISC proteins. *In vivo* interactions between both proteins were determined by measuring $\beta$-galactosidase activity (Rupp, 2002).

### Results

### Structural relationship between the yeast monothiol glutaredoxins Grx3, Grx4 and Grx5

The three monothiol glutaredoxins of *S. cerevisiae* differ in their structure. Grx5 contains a glutaredoxin (Grx) module that is fused to an N-terminal mitochondrial targeting sequence (Figure 1A) (Rodríguez-Manzaneque *et al.*, 2002). The Grx module is conserved in many organisms, from bacteria to humans (Bellí *et al.*, 2002). In the latter, it has been named PICOT homology domain, after the PICOT protein (a negative regulator of protein kinase C-$\theta$) (Witte *et al.*, 2000). Grx3 and Grx4 lack the mitochondrial targeting sequence, a fact that correlates with their non-mitochondrial location (as predicted by MITOPROT analysis) and their failure to rescue the phenotype resulting from Grx5 deletion (Rodríguez-Manzaneque *et al.*, 2002; Bellí *et al.*, 2002).

Grx3 and Grx4, but not Grx5, also contain an N-terminal thioredoxin (Trx) domain (Figure 1A). This domain is present in many eukaryotic homologues of Grx3/Grx4, including the PICOT protein, but it is absent in all their prokaryotic homologues (Bellí *et al.*, 2002). Closer analysis of the Trx domain extension (Figure 1B) shows that it is homologous to a significant part of the thioredoxin molecules, but it lacks the first cysteine residue that is characteristic of the thioredoxin active site (WCGPCK) and is essential for thioredoxin-mediated dithiol protein reduction (Holmgren, 1989; Powis and Montfort, 2001). However, other amino acids in the active site region, including the second cysteine, are conserved when compared with the three genuine thioredoxins of *S. cerevisiae* (Figure 1B). The above observations strongly suggest that Grx3/Grx4 and their eukaryotic homologues derive from the fusion of a thioredoxin domain to a monothiol Grx module in an eukaryotic ancestor after separation from prokaryotes. Comparing the amino acid sequences from the Trx module in Grx3 and Grx4 with those from a number of genuine thioredoxin molecules provides further support for this prediction. The maximum parsimony phylogenetic consensus tree (calculated using PHILIP; Felsenstein, 1993) for the ClustalW alignment (Figure 1C) shows that the Trx module of the two yeast glutaredoxins has diverged at an early stage from all the eukaryotic genuine thioredoxins included in the analysis. This further supports an early separation of the Grx3/Grx4 Trx module from the other thioredoxin domains within the eukaryotic line, by fusion of Trx to an ancestor monothiol glutaredoxin and separate evolution. The fact that no bacterial species contains molecules with the Trx–Grx structure argues against the presence of this type of molecule in the endosymbiotic ancestor of eukaryotes.

### Comparison of prokaryotic and eukaryotic monothiol glutaredoxins

We extended the comparison of proteins containing the monothiol Grx domain characteristic of Grx5 to a total of 26 proteins from the NCBI and KEEG databases. Thirteen of these were prokaryotic (12 bacteria plus one archaean, all of them with a single Grx domain not fused to any Trx domain), and 13 were eukaryotic. Among the latter, seven sequences contained an additional Trx domain in the N-region. Only the Grx domains without extensions were used to generate a tree after ClustalW alignment (Figure 2). In parallel, we predicted the cellular location for each of the eukaryotic protein sequences. All the eukaryotic proteins with the Trx–Grx structure (included Grx3 and Grx4) group together in the tree and none of them is predicted to be mitochondrial.

Grx5 is in a separate group from the eukaryotic Trx–Grx molecules, together with homologues from other eukaryotic organisms (Figure 2). The group includes the only sequenced archaean that has a monothiol glutaredoxin (*Halobacterium* sp.). With the exception of the latter, all the proteins grouped in this cluster have a single monothiol Grx domain and are predicted to be in the mitochondria.

Copyright © 2004 John Wiley & Sons, Ltd. 

It is significant that the monothiol glutaredoxin of the $\alpha$-proteobacterium *Rickettsia prowazekii*, proposed to be the closest fully sequenced relative to the endosymbiotic ancestor of mitochondria (Andersson *et al.*, 1998), has separated early from all other sequences (Figure 2).

The above results further support the idea that duplication of an ancestor monothiol glutaredoxin gene, followed by fusion of a Trx module to one of the duplicated Grx genes, occurred early during eukaryotic evolution after endosymbiosis, probably with a *Rickettsia* ancestor. This would explain why molecules with the Trx–Grx structure are found in eukaryotic groups that range from unicellular fungi to plants and mammals. The absence of a Trx–Grx monothiol glutaredoxin gene in some eukaryotic fully sequenced genomes, such as that of *Caenorhabditis elegans*, could be explained by loss of the Trx domain at genome level rather than by repeated Trx–Grx fusion events in the eukaryotic lineage.

### Phylogenetic profiles of Grx5 homologues and other proteins involved in Fe–S cluster assembly

Based on the fact that Grx5 participates in the synthesis of Fe–S clusters at the mitochondrial matrix (Rodríguez-Manzaneque *et al.*, 2002), we studied the co-occurrence of Grx5 homologues with homologues of the other yeast mitochondrial proteins participating in the synthesis of the clusters. We used 67 completely sequenced prokaryotic chromosomes (51 bacteria plus 16 archaeans) for the analysis (Figure 3). Grx5 homologues are present in all the proteobacteria except the $\varepsilon$ group, in common with most of the other Fe–S-assembly proteins studied. Besides proteobacteria, only the two cyanobacteria species analysed plus *Halobacterium* sp., contain Grx5 homologues. Among the other Fe–S cluster synthesis-related proteins, Isa1 and Jac1 have the phylogenetic profiles that are more similar to that of Grx5, followed by Yfh1 and Nfs1 (Figure 3). On the contrary, Arh1 and Ssq1 gave the most disimilar patterns to Grx5.

Although Grx5 homologues are absent in many bacteria where there are Ssq1 homologues, Ssq1 homologues are always present in bacteria with Grx5 homologues. In *S. cerevisiae*, overexpression of Ssq1 rescue the defects caused by the lack of Grx5, which points to a close functional relationship between Ssq1 and Grx5 in yeast cells (Rodríguez-Manzaneque *et al.*, 2002). Furthermore, low-definition protein docking experiments predict that these two molecules form specific protein complexes that may help to fold/stabilize Grx5 as a functional protein (see below).

The case of Arh1 (ferredoxin reductase) is different from that of Ssq1. Arh1 homologues are found in many organisms where no Grx5 homologues are found and vice versa. This indicates that Arh1 functional homologues are not necessarily sequence homologues, suggesting a case of convergent evolution for the ferredoxin reductase activity between different organisms. To further support this, it is known that Arh1 in *S. cerevisiae* is a sequence homologue to the eukaryotic NADP-dependent adrenodoxin reductase (Manzella *et al.* 1998) while in many bacteria there is no such homology. For example, the *Buchnera* Arh1 functional homologue (gene BU581, SwissProt entry P57641) has no sequence similarity to the *S. cerevisiae* Arh1 ferredoxin reductase, according to the Iteralign algorithm (Brocchieri and Kerlin, 1998).

As a control, in the study we included three mitochondrial proteins of *S. cerevisiae* that are not involved in Fe–S cluster synthesis. Trx3 is a mitochondrial thioredoxin (Pedrajas *et al.*, 1999), Cox1 is the cytochrome *c* oxidase subunit I, encoded by a mitochondrial gene (Lemaire *et al.*, 1998), and Pdb1 is the pyruvate dehydrogenase $\beta$-subunit (Miran *et al.*, 1993). Homologues of these three proteins exist in a wider range of prokaryotic microorganisms than Grx5 homologues (Figure 3), even in the case of a mitochondrially-encoded protein such as Cox1. This shows that the phylogenetic profile of a protein is not necessarily determined by the mitochondrial character of the protein.

The $\gamma$-proteobacterium *Buchnera* sp. contains the smallest fully sequenced genome (Shigenobu *et al.*, 2000) that encodes for a Grx5 homologue (Figure 3). Therefore, we made a PSI-BLAST comparison of the products of the 564 coding genes of *Buchnera* sp. APS against all the proteins from 79 genomes (55 bacteria, 14 archaeans and 10 eukaryotes) deposited in non-overlapping KEGG and NCBI databases. For each protein pair formed by the *Buchnera* sp. Grx5 homologue (gene BU187) and any other protein from this bacterium, we calculated the index of co-occurrence, i.e. the fraction of organisms in which homologues of both proteins are simultaneously present

Copyright © 2004 John Wiley & Sons, Ltd.

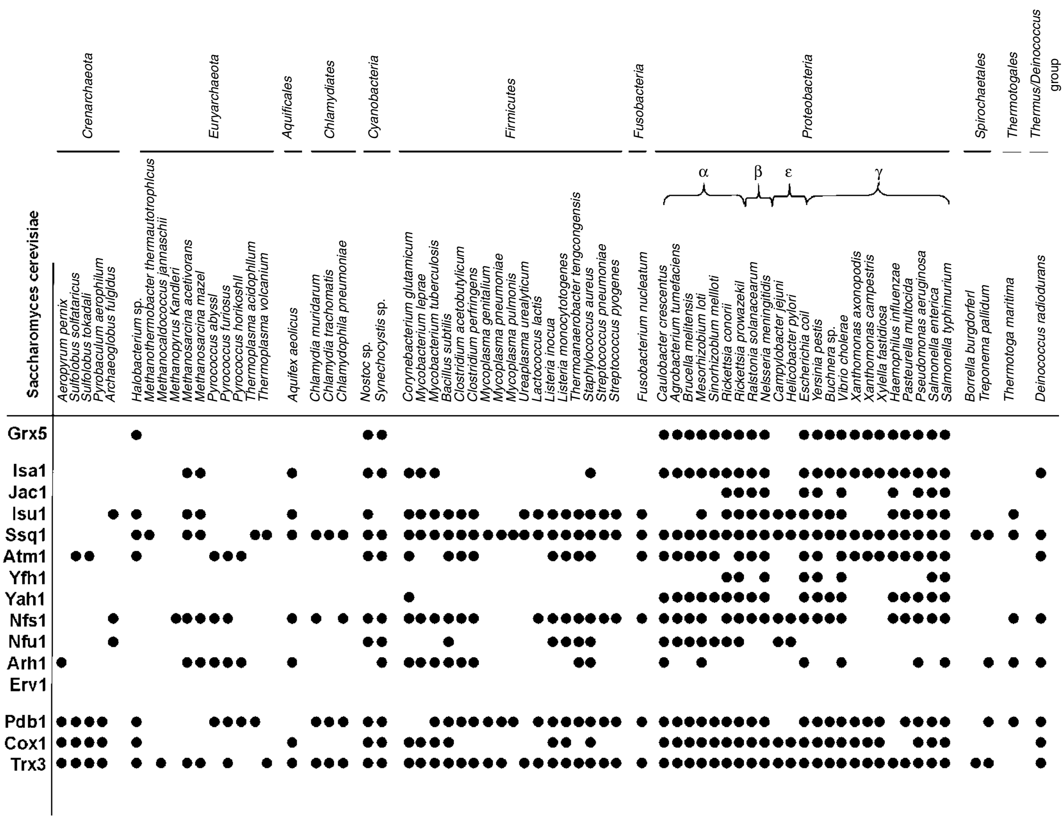

**Figure 3.** Phylogenetic distributions among prokaryotic species of homologues of Grx5 glutaredoxin and of other *S. cerevisiae* mitochondrial proteins involved in the assembly of Fe–S clusters. Only organisms with fully sequenced genomes were included in the analysis. The left column indicates the names of the *S. cerevisiae* proteins. Yeast mitochondrial proteins not participating in Fe–S cluster synthesis are also shown (bottom three rows). Dots mark the presence of a homologue in the respective prokaryotic organism. Homology was determined by PSI-BLASTA searches in the NCBI database, as detailed in Materials and methods or absent. Five *Buchnera* sp. proteins have an index of co-occurrence with Grx5 higher that 0.85. Two of them are the homologues of the Yah1 ferredoxin and Isa1, respectively. These and the above results therefore support the idea that, among bacteria, a number of Fe–S cluster biogenesis-related proteins, particularly the Yah1 and Isa1 orthologues, have closely co-evolved with the Grx5 orthologue.

The determination of the index of co-occurrence, referred to Grx5 and its homologues, was extended to all the *S. cerevisiae* open reading frame products and the respective homologues present in the same 79 genomes as above. An index of 0.8 or higher was obtained for 52 *S. cerevisiae* proteins. It is to be expected that proteins functionally interacting with Grx5 would share cellular location. Therefore, only the subset of mitochondrial proteins was further considered. This leaves a total of 24 yeast mitochondrial proteins with an index of co-occurrence with Grx5 above the 0.8 threshold (Table 1). Among these, five are involved in the assembly of Fe–S clusters: Yah1, Isa1, Isa2, Nfu1 and Yfh1. Remarkably, the first four of these proteins display a high co-occurrence pattern with Yfh1, as shown in a previous study (Huynen *et al.*, 2001). The results reinforce the proposed role of Grx5 in the assembly of Fe–S clusters (Rodríguez-Manzaneque *et al.*, 2002), in close relationship with a subset of proteins that includes the Yfh1 frataxin. Two other proteins that also have a high index of co-occurrence, as shown in Table 1, are Oct1 and Prd1. These are functionally redundant mitochondrial intermediate peptidases that participate in the final maturation of imported peptides in the mitochondria (Isaya *et al.*, 1994; Neupert, 1997). The importance of Oct1 in the maturation

Copyright © 2004 John Wiley & Sons, Ltd. 

**Table 1.** Index of co-occurrence between homologues of Grx5 and *S. cerevisiae* mitochondrial proteins

| *S. cerevisiae* ORF | Protein | Index of co-occurrence | Known or predicted function[a] |
|---|---|---|---|
| YAL044w-A | Unknown | 0.975 | Putative DNA repair protein |
| YKL134c | Oct1 | 0.925 | Mitochondrial import protein |
| YPL252 | Yah1 | 0.912 | Ferredoxin, Fe–S assembly |
| YLR239c | Lip2 | 0.900 | Lipoyl ligase |
| YDR044w | Hem13 | 0.900 | Heme biosynthetic pathway |
| YGR255c | Coq6 | 0.887 | Monooxygenase, ubiquinone biosynthesis |
| YPR067w | Isa2 | 0.862 | Fe–S assembly |
| YMR118c | Unknown | 0.862 | Unknown |
| YCL057w | Prd1 | 0.862 | Metalloendopeptidase |
| YOR065w | Cyt1 | 0.850 | Cytochrome c1 subunit |
| YMR193w | MrpL24 | 0.850 | Mitochondrial ribosomal protein |
| YLR316c | Tad3 | 0.850 | tRNA-specific adenosine deaminase subunit |
| YLL027w | Isa1 | 0.850 | Fe–S assembly |
| YEL052w | Afg1 | 0.850 | ATPase |
| YDL004w | Atp16 | 0.850 | Hydrogen-transporting ATPase |
| YKR087c | Unknown | 0.837 | Unknown |
| YKL040c | Nfu1 | 0.837 | Fe–S assembly |
| YGL136c | Mrm2 | 0.837 | rRNA methyltransferase |
| YMR234w | Rnh1 | 0.825 | Ribonuclease H |
| YLR059c | Rex2 | 0.825 | 3′–5′ RNA exonuclease |
| YPL132w | Cox11 | 0.812 | Assembly of cytochrome *c* oxidase |
| YKL141w | Sdh3 | 0.800 | Succinate dehydrogenase |
| YDL120W | Yfh1 | 0.800 | Frataxin, Fe–S assembly |
| YBR026c | Mrf1 | 0.800 | Enoyl–(acyl-carrier protein) biosynthesis |

[a] From the *Saccharomyces* Genome Database.

of the Fe–S-containing protein cytochrome $bc_1$ has been shown (Isaya and Kalousek, 1995; Nett *et al.*, 1997), as well as the functional interaction between Oct1 and the Yfh1 frataxin (Branda *et al.*, 1999). Altogether, the results suggest that Oct1/Prd1 could be important for Grx5 maturation/import into mitochondria.

### Protein interaction prediction between Grx5 and proteins involved in Fe–S cluster assembly

Determining the existence of strong specific protein complexes between Grx5 and other components of the ISC machinery in yeast would support the proposed role of Grx5 and establish hierarchical relationships with a subset of these components. Because there is no available three-dimensional structure (determined by nuclear magnetic resonance or X-ray crystallography analyses) for any of the Fe–S cluster synthesis proteins of *S. cerevisiae*, we used 3D-JIGSAW (Bates *et al.*, 2001) to obtain predictions for these structures. We then used low definition protein docking (Vakser and Jiang, 2001) to predict the strength and stability/specificity of the strongest complexes between Grx5 and other ISC proteins (Table 2). Highest values for complex strength and specificity were obtained for Grx5 interactions with Arh1, Ssq1 and Isa1. Importantly, these values were comparable with the positive control between Yah1 and its reductase Arh1 (Table 2). We made another test to investigate the validity of the algorithms used. Bovine ferredoxin and ferredoxin reductase form a complex that has been determined by X-ray crystallography (Protein Database, entry No. 1E6E). We applied the GRAMM docking algorithm to the two separate proteins and recovered the same complex as the experimentally determined one (data not shown). A final positive control was done by docking the signal peptide of yeast malate dehydrogenase to its processive protease and recovering a complex that is approximately the crystallized one (Protein Database, entry No. 1HR9).

Other proteins showing high evolutionary co-occurrence with Grx5 in Table 1 were also analysed for their interactions with this glutaredoxin.

Copyright © 2004 John Wiley & Sons, Ltd. 

**Table 2.** Prediction of complex strength and complex specificity/stability between Grx5 and other Fe–S cluster biosynthesis proteins or mitochondrial proteins with high index of co-occurrence

| Protein complex | Complex strength[a] | Complex specificity[b] |
|---|---|---|
| Grx5/Ssq1 | 517 | 14 |
| Grx5/Arh1 | 658 | 15 |
| Grx5/Isa1 | 389 | 12 |
| Grx5/Atm1 | 497 | 7 |
| Grx5/Jac1 | 471 | 6 |
| Grx5/Nfu1 | 376 | 2 |
| Grx5/Isa2 | 343 | 5 |
| Grx5/Isu1 | 334 | 5 |
| Grx5/Yah1 | 326 | 5 |
| Grx5/Yfh1 | 325 | 3 |
| Arh1/Yah1 | 615 | 17 |
| Grx5/Oct1 | 1068 | 13 |
| Grx5/Prd1 | 1024 | 7 |
| Grx5/Rex2 | 497 | 2 |
| Grx5/Cyt1 | 468 | 4 |
| Grx5/Lip2 | 428 | 3 |
| Grx5/Atp16 | 370 | 2 |
| Grx5/Cox11 | 321 | 4 |

[a] The complex strength is measured in arbitrary and consistent energy units (Vakser and Jiang, 2001).
[b] The complex specificity is measured as the number of instances in which each protein pair appears within the 20 strongest complexes having approximately the same coordinates for the interacting surface.

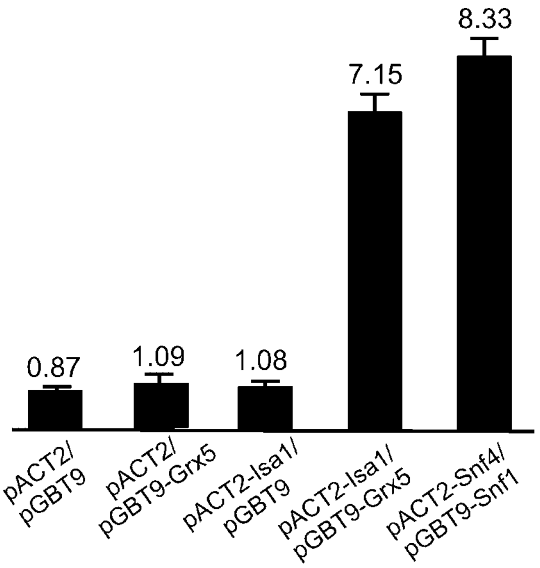

**Figure 4.** Two-hybrid analysis of the interaction between Grx5 and Isa1. Numbers over bars indicate the $\beta$-galactosidase activity (Miller units) in cultures of *S. cerevisiae* cells co-transformed with the indicated plasmids: pGBT9 and pACT2 vectors alone, or derivatives expressing the respective Gal4 fusion proteins with Grx5, Isa1, Snf1 or Snf4. The results are the means of three independent experiments

Among these, only complexes with Oct1 or Prd1 are predicted to be both strong and stable/specific (Table 2), with Oct1 giving the highest score. Altogether, the results support the direct involvement of Oct1/Prd1 in Grx5 import/maturation. As negative controls, we included the strength and specificity parameters of the respective pairs formed by Grx5 and each of five mitochondrial proteins (Rex2, Cyt1, Lip2, Atp16 and Cox11), which are not related with Fe–S cluster biosynthesis (Table 2).

### Two-hybrid analyses confirm the physical interaction between Grx5 and Isa1

In order to confirm the predicted interaction between Grx5 and other ISC components, we carried out directed two-hybrid analyses involving Grx5 (fused to the DNA binding domain of Gal4) and any of the other known ISC components as the partner fused to the Gal4 activator domain. Using $\beta$-galactosidase activity as reporter for in vivo interactions, we observed positive interaction of Grx5 with Isa1 (Figure 4), but not with other ISC components (not shown). It is remarkable that with the above assay, the Grx5/Isa1 interaction was almost as strong as the well known interaction between Snf1 and Snf4 proteins that was used as a positive control. The negative results with other ISC components could reflect the absence of other real *in vivo* interactions, or rather the fact that in the present assay only interactions that are stable in the cell nucleus are readily detected. In any case, our results confirm that Grx5 interacts in vivo with some ISC components.

### Discussion

Of the three monothiol glutaredoxins in yeast, Grx5 participates in the formation of iron–sulphur (Fe–S) clusters in the mitochondria (Bellí *et al.*, 2002), together with other mitochondrial proteins (Lill and Kispal, 2000; Mühlenhoff and Lill, 2000; Mühlenhoff *et al.*, 2002). These include Nfs1, a cysteine desulphurase that provides sulphur to the Fe–S biosynthetic machinery, as well as Isu1/Isu2 and Isa1/Isa2 (two pairs of proteins that act as

Copyright © 2004 John Wiley & Sons, Ltd.

scaffolds for the assembly of iron and sulphur into the clusters), and the chaperone Ssq1 and its co-chaperone Jac1 (respectively from the Hsp70 and Hsp40 types). The process of Fe–S synthesis requires NADH as electron donor (Mühlenhoff *et al.*, 2002), through a transport chain involving the ferredoxin Yah1 and the ferredoxin reductase Arh1. Nfu1 is another mitochondrial protein with a non-characterized partially dispensable role in the Fe–S cluster assembly. The participation of the frataxin Yfh1 protein in the biogenesis of the clusters is object of discussion. Initially, Yfh1 was proposed to participate in the cellular homeostasis of iron because the lack of Yfh1 leads to iron accumulation in the mitochondria (Babcock *et al.*, 1997). More recent biochemical studies, however, support a direct role of frataxin in the Fe–S cluster assembly, indicating that the mitochondrial accumulation of iron is a secondary consequence of the disruption of the iron assembly into the Fe–S clusters (Chen *et al.*, 2000; Mühlenhoff *et al.*, 2002). Although synthesis of all the Fe–S clusters occurs at the mitochondrial matrix in yeast, two additional proteins are required for the assembly of the clusters into extramitochondrial proteins: Atm1 is an ABC transporter located at the inner mitochondrial membrane (Kispal *et al.*, 1999), while the mitochondrial intermembrane space protein Erv1 would operate downstream of Atm1 in the export of the Fe–S clusters destined to the cytosolic or nuclear apoproteins (Lange *et al.*, 2001). The absence of Grx5 causes phenotypes similar to those caused by the absence of other yeast proteins participating in the synthesis of the clusters, among them defects in the activity of Fe–S enzymes, the incapacity for respiratory growth and the accumulation of iron in the cell (Rodríguez-Manzaneque *et al.*, 2002).

The above proteins constitute the so-called ISC machinery, and many of them have orthologues in prokaryotes, which shows that the process of Fe–S cluster formation arose early in evolution (Mühlenhoff and Lill, 2000). In bacteria, most of the genes involved are grouped in a single *isc* operon. *Azotobacter vinelandii* possesses a second *nif* operon that codes for proteins specifically involved in the formation of the Fe–S cluster of the nitrogenase (NIF machinery) (Peters *et al.*, 1995).

No detailed biochemical data exist supporting the idea that Fe–S cluster assembly in *S. cerevisiae* occurs in a multiprotein complex involving several or all of the ISC proteins. However, the fact that the *NFU1* gene has been detected in a synthetic lethal screen with *SSQ1* leads to hypothesize that Nfu1 is a direct or indirect substrate of the Ssq1 chaperone (Schilke *et al.*, 1999). Moreover, physical interaction between Isu1 and Nfs1 has been revealed in a comprehensive mass-spectrometry study of protein–protein interactions in yeast (Ho *et al.*, 2002), in experimental conditions that support the biological relevance of such interaction. Studies on Fe–S cluster synthesis in *Escherichia coli* suggest that stable protein–protein interactions are established among components of the ISC machinery (Agar *et al.*, 2000). According to those studies, IscU (*E. coli* orthologue of Isu1) acts as an scaffold for the ISC components (Agar *et al.*, 2000). In particular, IscS (Nfs1) participates at an early stage of the Fe–S cluster assembly as a cysteine desulphurase that transfers a sulphur atom from L-cysteine to IscU and then to the Fe–S cluster, in a process where a covalently-bound IscS/IscU complex is formed (Urbina *et al.*, 2001; Smith *et al.*, 2001; Kato *et al.*, 2002). The process is specifically assisted by the Hsc66 (Ssq1) chaperone and the Hsc20 (Jac1) co-chaperone (Silberg *et al.*, 2001; Hoff *et al.*, 2002). On the other hand, based on physical chemistry studies, IscA (Isa1) has been proposed as an alternative scaffold to IscU (Krebs *et al.*, 2001) and it forms a functional stable complex with the *E. coli* ferredoxin (Ollagnier-de-Choudens *et al.*, 2001).

The yeast Grx5 protein is a model for monothiol glutaredoxins formed by a single Grx domain. Homologous proteins with the same domain architecture are present both in prokaryotes and eukaryotes, although the only homologue with a known functional role is Grx5. This paper shows that the phylogenetic profile of Grx5 homologues among prokaryotes is similar to the profiles of a subset of proteins of the ISC machinery, suggesting that prokaryotic Grx5 orthologues are also required for the assembly of the Fe–S complexes. Among bacteria, the ISC machinery is characteristically present in the $\alpha$-, $\beta$- and $\gamma$-proteobacteria groups (Mühlenhoff and Lill, 2000). Homologues of Grx5 are present in those bacterial groups, but absent in other prokaryotes for which fully sequenced genomes exist, with the exceptions of cyanobacteria and *Halobacterium* sp. The presence of a Grx5 homologue in this archaean may reflect the high frequency of horizontal gene transfer between *Halobacterium* and bacteria (Korbel *et al.*, 2002).

Copyright © 2004 John Wiley & Sons, Ltd. 

Although Archaea species contain proteins with Fe–S clusters, these are likely to be synthesized by a third mechanism (in addition to the ISC and NIF machineries) recently characterized (Takahashi and Yokumoto, 2002). $\varepsilon$-Proteobacteria do not contain homologues of most of the ISC proteins, including Grx5 (Figure 3). In this bacterial group a NIF-like machinery seems to be responsible for the synthesis of the Fe–S clusters (Olson *et al.*, 2000).

Our phylogenetic profile studies extend those of Huynen *et al.* (2001), which were done for Yfh1 frataxin. The present work indicates that Grx5 homologues have not co-evolved in parallel with all the ISC machinery components in all organisms, pointing to functional specializations. The highest indexes of co-occurrence (larger than 0.80) are observed for Yah1, Isa1, Isa2, Nfu1 and Yfh1, in accordance with a previous study that supported co-evolution of Yfh1 with Yah1, Isa1/2 and Nfu1 (Huynen *et al.*, 2001). In addition, for Jac1, an index higher than 0.77 was obtained in our study (data not shown).

The results reported here predict a strong and stable interaction of Grx5 with Ssq1 and Arh1 (the reductase of Yah1) and a stable interaction with Isa1. We have confirmed that the latter interaction occurs *in vivo*, using two-hybrid analysis. The formation of the Fe–S clusters in the yeast mitochondrial matrix is not well understood, although the recent development of an *in vitro* assembly assay (Mühlenhoff *et al.*, 2002; 2003) may help in further elucidating this process. Nevertheless, based on the conservation of the function of the ISC machinery throughout evolution (Mühlenhoff *et al.*, 2000), results from *E. coli* and *A. vinelandii* studies (see Introduction and references therein) can be used to suggest the role of individual ISC components in yeast. Isa1 (maybe forming heterodimers with Isa2; Mühlenhoff *et al.*, 2000) could act as a scaffold for the initial assembly of the Fe–S clusters in a Nfs1-directed manner. The process could be assisted by the Ssq1/Jac1 chaperone. Once formed, the Fe–S clusters could be transferred to the Yah1 ferredoxin in a redox process in which Arh1 would be the NADPH-dependent reductase. The roles of Nfu1 and Yfh1 can not be predicted in this scenario, since their biochemical activities are unknown. Given the probable existence of a stable multiprotein complex where these reactions would occur, Grx5 would be part of this complex. As a monothiol glutaredoxin, it catalyses the deglutathionylation of mixed disulphides formed between glutathione and protein sulphydryl groups (Herrero and Ros, 2002). Grx5 could act (using a monothiol mechanism) in the reduction of disulphide bonds in the Isa proteins previous to coordination of iron atoms to the reduced cysteine residues of the protein. Alternatively, Grx5 could be necessary for repairing the inactivating mixed disulphides that could be formed, in oxidative conditions, between glutathione and the cysteine residues responsible for iron chelation. The destabilizing action of glutathione on Fe–S clusters has been shown in *E. coli* (Ding and Demple, 1996). Our theoretical and experimental results support the existence of a physical interaction of Grx5 with Isa1, but not with Isa2. It should be remarked that Isa1 and Isa2 do not necessarily carry out exactly overlapping functions (Rodríguez-Manzaneque *et al.*, 2002), which could be related to the fact that sequence homology between both proteins is restricted to some specific regions.

Other aspects of Fe–S cluster biosynthesis are emphasized by our results. First, import and processing of Grx5 into the mitochondria would require the activity of the Oct1 and Prd1 mitochondrial intermediate peptidases, which are predicted to form strong and specific interactions with Grx5. Second, an alternative role for Ssq1 (and Jac1) could be their participation in Grx5 folding during mitochondrial import, a role that could be extended to other ISC components. However, no data exist supporting this Ssq1 role during internalization. Third, although Arh1 is the Yah1 reductase in yeast (and this would explain the predicted interaction with Grx5), this is not a conserved trait along evolution. In fact, the index of co-occurrence between Arh1 and Grx5 homologues is rather low. A different ferredoxin reductase would operate in most bacteria.

Taken together, our results indicate that monothiol glutaredoxins with the simple domain structure characteristic of Grx5 were already present in primitive proteobacteria (except the $\varepsilon$-group), forming part of the ISC machinery. This function of monothiol glutaredoxins was transferred to early eukaryotes through the endosymbiotic ancestor, maintaining their function in the synthesis of Fe–S clusters, at least in yeast species. Besides Grx5, *S. cerevisiae* cells contain two more proteins (Grx3 and Grx4) in which the monothiol glutaredoxin

Copyright © 2004 John Wiley & Sons, Ltd. 

domain (Grx) is fused to a N-terminal thioredoxin-like (Trx) domain. Homologues with these characteristics are present in many other eukaryotes, but not in prokaryotes. The limited number of fully sequenced eukaryotic genomes prevents the application of the phylogenetic profile approach to Grx3/Grx4. Sequence analyses support the idea that these Trx–Grx molecules resulted from the duplication, early in the eukaryotic lineage, of an ancestral *GRX* gene followed by the fusion of a *TRX* gene to one of the duplicated copies. The presence of two different Trx–Grx molecules such as Grx3 and Grx4 in *S. cerevisiae* would result from a more recent duplication event, specific to the evolutionary line of budding yeast, as the fission yeast and other eukaryotes contain a single Trx–Grx glutaredoxin. The early addition of a Trx module to a glutaredoxin molecule in eukaryotes would have allowed the functional diversification of these molecules from the original Grx function in the ISC machinery, paralleled by differential compartmentation. In fact, in the case of *S. cerevisiae*, overexpression of Grx3 or Grx4 does not suppress the defects of a null *grx5* mutant (Rodríguez-Manzaneque *et al.*, 2002), while when we target either Grx3 or Grx5 to the mitochondria of these *grx5* mutant cells the defects are rescued (our unpublished observations). Therefore, although Grx3 or Grx4 would potentially substitute for the Grx5 function in the ISC machinery, in fact they have separate functions from Grx5. The situation would be similar in other eukaryotes where Grx and Trx–Grx proteins coexist, although in separate compartments. It is interesting to note that the thioredoxin domains of these molecules contain a conserved cysteine in what originally was the thioredoxin active site (Figure 1B). This residue may have a regulatory on the activity of Trx–Grx5 molecules, although further work is needed to elucidate the function of these eukaryotic thioredoxin–glutaredoxin chimaeric proteins.

### Acknowledgements

R.A. was supported by a Fellowship SB2000-031 from the Spanish Ministerio de Educacion, Cultura y Deporte. M.R-M. received a Marie Curie Training Fellowship from the European Union. S.S received a fellowship from the Swedish Foundation for Strategic Research. This work was supported by the Spanish Ministerio de Ciencia y Tecnología (Project No. BMC2001-1213-C02-01) and the Generalitat de Catalunya (Project No. 2001SGR-00305) to E.H., and by the Swedish Research Council for Science and Technology (grant 2003-3189) to P.S.

### References

Agar JN, Krebs C, Frazzon J, *et al.* 2000. IscU as a scaffold for iron–sulphur cluster biosynthesis: sequential assembly of [2Fe–2S] and [4Fe–4S] clusters in IscU. *Biochemistry* **27**: 7856–7862.

Altschul SF, Madden TL, Schaffer AA, *et al.* 1997. Gapped BLAST and PSI-BLAST: a new generation of protein database search programmes. *Nucleic Acids Res* **25**: 3389–3402.

Andersson SGE, Zomorodipour A, Andersson JO, *et al.* 1998. The genome sequence of *Rickettsia prowazekii* and the origin of mitochondria. *Nature* **396**: 133–140.

Babcock M, De Silva D, Oaks R, *et al.* 1997. Regulation of mitochondrial iron accumulation by Yfh1, a putative homolog of frataxin. *Science* **276**: 1709–1712.

Bates PA, Kelley LA, MacCallum RM, Sternberg MJE. 2001. Enhancement of protein modelling by human intervention in applying the automatic programmes 3D-JIGSAW and 3D-PSSM. *Proteins* **5**(suppl): 39–46.

Bellí G, Polaina J, Tamarit J, *et al.* 2002. Structure-function analysis of yeast Grx5 monothiol glutaredoxin defines essential amino acids for the function of the protein. *J Biol Chem* **277**: 37 590–37 596.

Benson DA, Karsch-Mizrachi I, Lipman DJ, *et al.* 2001. GenBank. *Nucleic Acids Res* **30**: 17–20.

Branda SS, Yang ZY, Chew A, Isaya G. 1999. Mitochondrial intermediate peptidase and the yeast frataxin homolog together maintain mitochondrial iron homeostasis in *Saccharomyces cerevisiae*. *Hum Mol Genet* **8**: 1099–1110.

Brocchieri L, Karlin S. 1998. A symmetric-iterated multiple alignment of protein sequences. *J Mol Biol* **276**: 249–264.

Bushweller JH, Aslund F, Wuthrich K, Holmgren A. 1992. Structural and functional characterization of the mutant *Escherichia coli* glutaredoxin (C14-S) and its mixed disulphide with glutathione. *Biochemistry* **31**: 9288–9293.

Chen OS, Haemnway S, Kaplan J. 2002. Inhibition of Fe–S cluster biosynthesis decreases mitochondrial iron export: evidence that Yfh1 affects Fe–S cluster synthesis. *Proc Natl Acad Sci USA* **99**: 12 321–12 326.

Claros MG, Vincens P. 1996. Computational method to predict mitochondrially imported proteins and their targeting sequences. *Eur J Biochem* **241**: 779–786.

Ding H, Demple B. 1996. Glutathione-mediated destabilization *in vitro* of [2Fe–2S] centers in the SoxR regulatory protein. *Proc Natl Acad Sci USA* **93**: 9449–9453.

Draculic T, Dawes IW, Grant CM. 2000. A single glutaredoxin or thioredoxin is essential for viability in the yeast *Saccharomyces cerevisiae*. *Mol Microbiol* **36**: 1167–1174.

Ettema T, van der Oost J, Huynen MA. 2001. Modularity in the gain and loss of genes: applications for function prediction. *Trends Genet* **97**: 485–487.

Felsenstein J. 1993. PHYLIP (Phylogeny Inference Package) version 3.5c: **http://evolution.genetics.washington.edu/phylip.html**

Gaasterland T, Ragan M. 1998. Phyletic and functional patterns of ORF distribution among prokaryotes. *J Microb Comp Genom* **3**: 199–217.

Copyright © 2004 John Wiley & Sons, Ltd. 

Herrero E, Ros J. 2002. Yeast glutaredoxins and protection against oxidative stress. *Methods Enzymol* **348**: 136–146.

Ho Y, Gruhler A, Heilbut A, *et al*. 2002. Systematic identification of protein complexes in *Saccharomyces cerevisiae* by mass spectrometry. *Nature* **415**: 180–183.

Hoff KG, Ta DT, Tapley TL, Silberg JJ, Vickery LE. 2002. Hsc66 substrate specificity is directed toward a discrete region of the iron–sulphur cluster template protein IscU. *J Biol Chem* **277**: 27 353–27 359.

Holmgren A. 1989. Thioredoxin and glutaredoxin systems. *J Biol Chem* **254**: 13 963–13 966.

Holmgren A, Aslund F. 1995. Glutaredoxin. *Methods Enzymol* **252**: 283–292.

Huynen M, Snel B, Lathe W III, Bork P. 2000. Predicting protein function by genomic context: quantitative evaluation and qualitative inferences. *Genome Res* **10**: 1204–1210.

Huynen M, Snel B, Bork P, Gibson TJ. 2001. The phylogenetic distribution of frataxin indicates a role in iron–sulphur cluster protein assembly. *Hum Mol Genet* **10**: 2463–2468.

Isaya G, Miklos D, Rollins RE. 1994. *MIP1*, a new yeast gene homologous to the rat mitochondrial intermediate peptidase gene, is required for oxidative metabolism in *Saccharomyces cerevisiae*. *Mol Cell Biol* **14**: 5603–5610.

Isaya G, Kalousek F. 1995. Mitochondrial intermediate peptidase. *Methods Enzymol* **248**: 556–567.

Kato S, Mihara H, Kurihara T, *et al*. 2002. Cys-328 of IscS and Cys-63 of IscU are the sites of disulphide bridge formation in a covalently bound IscS/IscU complex: implications for the mechanism of iron–sulphur cluster assembly. *Proc Natl Acad Sci USA* **99**: 5948–5952.

Kispal C, Csere P, Prohl C, Lill R. 1999. The mitochondrial proteins Atm1p is required for mitochondrial iron homeostasis. *EMBO J* **18**: 3981–3989.

Korbel JO, Snel B, Huynen MA, Bork P. 2002. SHOT: a web server for the construction of genome phylogenies. *Trends Genet* **18**: 158–162.

Krebs C, Agar JN, Smith AD, *et al*. 2001. IscA, an alternate scaffold for Fe–S cluster biosynthesis. *Biochemistry* **40**: 14 069–14 080.

Lange H, Lisowsky T, Gerber J, *et al*. 2001. An essential function of the mitochondrial sulphydryl oxidase Erv1p/ALR in the maturation of cytosolic Fe–S proteins. *EMBO Rep* **2**: 715–720.

Lemaire C, Robineau S, Netter P. 1998. Molecular and biochemical analysis of *Saccharomyces cerevisiae cox1* mutants. *Curr Genet* **34**: 138–145.

Lill R, Kispal G. 2000. Maturation of cellular Fe–S proteins: an essential function of mitochondria. *Trends Biochem Sci* **25**: 352–356.

Luikenhuis S, Dawes IW, Grant CM. 1997. The yeast *Saccharomyces cerevisiae* contains two glutaredoxin genes that are required for protection against reactive oxygen species. *Mol Biol Cell* **9**: 1081–1091.

Manzella L, Barros MH, Nobrega FG. 1998. *ARH1* of *Saccharomyces cerevisiae*: a new essential gene that codes for a protein homologous to the human adrenodoxin reductase. *Yeast* **14**: 839–846.

Marcotte EM, Pellegrini M, Ng HL, *et al*. 1999. Detecting protein function and protein–protein interactions from genome sequences. *Science* **285**: 751–753.

Marcotte EM, Xenarios I, van der Bliek AM, Eisenberg D. 2000. Localizing proteins in the cell from their phylogenetic profiles. *Proc Natl Acad Sci USA* **97**: 12 115–12 120.

Miran SG, Lawson JE, Reed LJ. 1993. Characterization of *PDHβ1*, the structural gene for the pyruvate dehydrogenase $\beta$-subunit from *Saccharomyces cerevisiae*. *Proc Natl Acad Sci USA* **90**: 1252–1256.

Mühlenhoff U, Lill R. 2000. Biogenesis of iron–sulphur proteins in eukaryotes: a novel task of mitochondria that is inherited from bacteria. *Biochim Biophys Acta* **1459**: 370–382.

Mühlenhoff U, Richhardt N, Gerber J, Lill R. 2002. Characterization of iron–sulphur protein assembly in isolated mitochondria. *J Biol Chem* **277**: 29 810–29 816.

Mühlenhoff U, Gerber J, Richhardt N, Lill R. 2003. Components involved in assembly and dislocation of iron-sulfur clusters on the scaffold protein Isu1p. *EMBO J* **22**: 4815–4825.

Nett JH, Denke E, Trumpower BL. 1997. Two-step processing is not essential for the import and assembly of functionally active iron–sulphur protein into the cytochrome $bc_1$ complex in *Saccharomyces cerevisiae*. *J Biol Chem* **272**: 2212–2217.

Neupert W. 1997. Protein import into mitochondria. *Annu Rev Biochem* **66**: 863–917.

Ollagnier-de-Choudens S, Mattioli T, Takahashi Y, Fontecave M. 2001. Iron–sulphur cluster assembly: characterization of IscA and evidence for a specific and functional complex with ferredoxin. *J Biol Chem* **276**: 22 604–22 607.

Olson JW, Agar JN, Johnson MK, Maier RJ. 2000. Characterization of the NifU and NifS Fe–S cluster formation proteins essential for viability in *Helicobacter pylori*. *Biochemistry* **39**: 16 213–16 219.

Pedrajas JR, Kosmidou E, Miranda-Vizuete A, *et al*. 1999. Identification and functional characterization of a novel mitochondrial thioredoxin system in *Saccharomyces cerevisiae*. *J Biol Chem* **274**: 6366–6373.

Pellegrini M, Marcotte EM, Thompson MJ, Eisenberg D, Yeates TO. 1999. Assigning protein functions by comparative genome analysis: protein phylogenetic profiles. *Proc Natl Acad Sci USA* **96**: 42 885–42 888.

Peters JW, Fisher K, Dean DR. 1995. Nitrogenase structure and function: a biochemical–genetic perspective. *Annu Rev Microbiol* **49**: 335–366.

Powis G, Montfort WR. 2001. Properties and biological activities of thioredoxins. *Annu Rev Pharmacol Toxicol* **41**: 261–295.

Rahlfs S, Fischer M, Becker K. 2001. *Plasmodium falciparum* possesses a classical glutaredoxin and a second, glutaredoxin-like protein with a PICOT homology domain. *J Biol Chem* **276**: 37 133–37 140.

Rodríguez-Manzaneque MT, Ros J, Cabiscol E, Sorribas A, Herrero E. 1999. Grx5 glutaredoxin plays a central role in protection against protein oxidative damage in *Saccharomyces cerevisiae*. *Mol Cell Biol* **19**: 8180–8190.

Rodríguez-Manzaneque MT, Tamarit J, Bellí G, Ros J, Herrero E. 2002. Grx5 is a mitochondrial glutaredoxin required for the activity of iron/sulphur enzymes. *Mol Biol Cell* **13**: 1109–1121.

Rodríguez-Navarro S, Llorente B, Rodríguez-Manzaneque MT, *et al*. 2002. Functional analysis of yeast gene families involved in metabolism of vitamins $B_1$ and $B_6$. *Yeast* **19**: 1261–1276.

Rupp S. 2002. *LacZ* assays in yeast. *Methods Enzymol* **350**: 112–131.

Schilke B, Voisine C, Beinert H, Craig E. 1999. Evidence for a conserved system for iron metabolism in the mitochondria

Copyright © 2004 John Wiley & Sons, Ltd. 

of *Saccharomyces cerevisiae*. *Proc Nat Acad Sci USA* **96**: 10 206–10 211.

Shigenobu S, Watanabe H, Hattori M, Sakaki Y, Ishikawa H. 2000. Genome sequence of the endocellular bacterial symbiont of aphids *Buchnera* sp. APS. *Nature* **407**: 81–86.

Silberg JJ, Hoff KG, Tapley TL, Vickery LE. 2001. The Fe–S assembly protein IscU behaves as a substrate for the molecular chaperone Hsc66 from *Escherichia coli*. *J Biol Chem* **276**: 1696–1700.

Smith AD, Agar JN, Johnson KA, *et al*. 2001. Sulphur transfer from IscS to IscU: the first step in iron–sulphur cluster biosynthesis. *J Am Chem Soc* **123**: 11 103–11 104.

Takahashi Y, Tokumoto U. 2002. A third bacterial system for the assembly of iron–sulphur clusters with homologs in Archaea and plastids. *J Biol Chem* **277**: 28 380–28 383.

Thompson JD, Higgins DG, Gibson TJ. 1994. ClustalW: improving the sensitivity of progressive multiple sequence alignment through sequence weighting, position specific gap penalties and weight matrix choice. *Nucleic Acids Res* **22**: 4673–4680.

Urbina HD, Silberg JJ, Hoff KG, Vickery LE. 2001. Transfer of sulphur from IscS to IscU during Fe–S cluster assembly. *J Biol Chem* **276**: 44 521–44 526.

Vakser IA, Jiang S. 2001. Strategies for modeling the interactions of transmembrane helices of G protein-coupled receptors by geometric complementarity using the GRAMM computer algorithm. *Methods Enzymol* **343**: 313–28.

Witte S, Villalba M, Bi K, Liu Y, Isakov N, Altman A. 2000. Inhibition of the C-Jun N-terminal kinase/AP-1 amd NF-$\kappa$B pathways by PICOT, a novel protein kinase C-interacting protein with a thioredoxin homology domain. *J Biol Chem* **275**: 1902–1909.

Copyright © 2004 John Wiley & Sons, Ltd. 
